# The pollution indices of trace elements in soils and plants close to the copper and zinc smelting works in Poland’s Lower Silesia

**DOI:** 10.1007/s11356-020-08072-0

**Published:** 2020-02-25

**Authors:** Anna Hołtra, Dorota Zamorska-Wojdyła

**Affiliations:** Faculty of Environmental Engineering, Wrocław University of Science and Technology, Wybrzeże Wyspiańskiego 27, 50-370 Wrocław, Poland

**Keywords:** Toxic elements, Soil quality, Contamination factor, Geoaccumulation index, Degree of contamination, Bioaccumulation index

## Abstract

The quality of soils polluted by trace elements around the facilities with the Cu and Zn smelting activities and the post-flotation tailings pond from copper industry were assessed. The level of the contamination of soils was determined on the basis of the contamination factor and the geoaccumulation index. The geoaccumulation index allows to distinguish more degrees of soil contamination and simplifies the assessment of the useable value of soil. The degrees of soil contamination and the pollution load index were shown. It has been shown that the pollution indices are a useful tool in describing the soil quality and planning corrective actions in the areas contaminated as a result of industrial activity. Histograms of pollution indices were used in order to detect the distribution of trace elements in soils. The content of metals in biomass was assessed using bioaccumulation indices. *Triticum* L. and *Brassica napus* L. show low bioaccumulation of studied metals in cereal plants. The correlations were used in order to detect the relationship among trace elements in soil as well as the relationship of metal (soil)-metal (plant) and metal bioaccumulation (plant)-metal (soil). The highest values of indices were recorded for the Oława smelter, presumably due to the long operation period before technological changes limiting the emission of pollutants were introduced. This research area was classified as very highly contaminated with all trace elements. Soils around other facilities are at least moderately contaminated.

## Introduction

The metallurgical industry of non-ferrous metals is particularly oppressive to the environment and human health (Mukhacheva 2017; Kim et al. 2016; Kalinovic et al., 2016; Jamshidian et al. 2015). In Poland, this industry dynamically develops mainly in Upper and Lower Silesia, significantly changing the quality of the environment near the smelters (Kapusta and Sobczyk 2015; Skubała and Zaleski 2012; Damek-Poprawa and Sawicka-Kapusta 2003). Many authors confirm that long-term emission of gaseous pollutants and metal-bearing dusts is the source of the enrichment of soils with metals around the emitter of pollutants. The impact of copper smelters was considered by Vorobeichik and Kaigorodova (2017); Nikolić et al. (2011); Medyńska-Juraszek and Kabała (2010); Kabała et al. (2008); Martley et al. (2004); and Adamo et al. (2002). Strong influence of zinc smelters on the environment was reported by Cuske et al. (2013); Filzek (2004); Goodarzi et al. (2002); and Sterckeman et al. (2000 and 2002). An additional source of the emission of pollutants into the environment is the landfills of slag from the ore smelting process and the tailings pond with the post-flotation wastes of ores (Karczewska et al., 2017, Kalinovic et al. 2016; Medyńska-Juraszek and Kabała 2012; Medyńska et al. 2009; Cabala et al. 2009; Krzaklewski et al. 2004).

The richest deposits of copper in Europe occur in the Legnica-Głogów Copper District, in the Lower Silesia region of Poland. All mines located here extract both sandstone ore and shale-carbonate ore with the copper mineralization—a series of Permo-Triassic sediments. Copper mineralization occurs mainly in the form of small grains of sulfides, most often distributed evenly, but in some places concentrated in the form of smudges and extended pockets. Locally, there are coarse-grain forms of mineralization in the shape of veins of varying thicknesses or irregularly located pockets. Simple copper sulfides: chalcocite and digenite predominate in these types of copper ores. The accompanying chemical elements are lead, silver, cobalt, zinc, and nickel. The average copper content is 1.9% of Cu, while the average silver content is equal to 61 g/Mg (Barlett et al. 2013). The copper ores are sent to the nearby the Głogów I, the Głogów II, and the Legnica smelters. The wastes after flotation of copper ores with a mass of up to 26 million Mg/year are deposited in the Żelazny Most reservoir. The ongoing reconstruction of the facility is aimed at achieving a capacity of 1.1 billion m^3^. The Legnica copper smelter has been in operation since 1953 and is located on the outskirts of the city, about 4 km from the city center. It uses the shaft furnace technology and its production capacities of the electrolytic copper amount to approx. 111.1 thousand Mg/year. The smelter is surrounded with agricultural areas where mainly wheat and rape are grown. The Głogów copper smelter comprises two branches. Since 1971, the Głogów I smelter was operated with the traditional technology of shaft furnaces with production capacity of 160 thousand Mg Cu/year. Between 2014 and 2016, the existing installation was replaced with the flash furnace and electric furnace technology. The Głogów II smelter commenced operation in 1978 with a flash furnace installation with production capacity of 150 thousand Mg Cu/year. In 1999, a protection zone around the Głogów copper smelter was established and the recultivation of lands was carried out. The zone was liquidated in 2005 and currently, the agricultural production near the smelter includes mainly the cultivation of wheat, potatoes, and sugar beets. The Oława zinc smelter has been operating since 1845 and is the largest producer of cadmium, lead, and zinc oxides. It is located in the south-eastern part of the city in the immediate vicinity of family houses and blocks of flats. At a distance of less than 1 km to the west from the site, the city park is located, followed by arable lands and garden plots. Maize, rapeseed, wheat, rye, potatoes, and sugar beets are the main crops growing there.

In assessing the content of trace elements in the soil, the index of geoaccumulation, the contamination factor, and the degree of metal contamination are most commonly used. These indicators are used to assess the intensity of anthropogenic pollutants deposition in surface layers of soil (Kowalska et al. 2018). It is possible to assume the global background coverage for the studied chemical element, referring to the average content of the element in the earth’s crust (Yaylali-Abanuz 2011; Loska et al. 2004; Tylor and McLennan 1995). The reference level of the chemical element may also mean the world average concentration of the metal in the clayey sediment, the so-called average shale value (Zahran et al. 2015; Rahman et al. 2012; Muller 1969). Rubio et al. (2000) recommend using a regional background because significant differences in the chemical composition of the soil can occur locally. Depending on the parent material, the metal concentration in soil can change even up to 2–3 rows of magnitude according to Blaser et al. (2000). Furthermore, Blaser et al. (2000) and Sutherland (2000) recognized that the concentration of elements from the deeper layers of the soil profile can be considered the local background for the upper soil layers. Manna and Maiti (2018) assumed as the background the content of metals in the soil sample from the uncontaminated area outside of the influence of anthropogenic activity.

This study aims at as follows: (1) assessment of the condition of soils in the immediate vicinity of the copper smelters in Legnica and Głogów and the post-flotation copper ore tailings pond, and the zinc Oława smelter in Lower Silesia, Poland, (2) assessment of the suitability of soils for the cultivation of cereal plants, and (3) assessment of the utility of pollution indices for the comprehensive evaluation of the degree of soil contaminated with metals.

## Materials and methods

### Sample collection and analytical procedure

Environmental samples were collected in June and July 2017. The sampling points were located at a distance of up to 2.5 km from the borders of industrial facilities: the copper and zinc smelters and the Żelazny Most reservoir with the post-flotation copper ore tailings from the mines and the ore enrichment facilities of KGHM Polish Copper Inc. The samples were taken from the surface soil layer up to the depth of 20 cm (plowing layer, green areas: city park, lawns, roadsides). The research material was collected in thirty not equally distributed sampling points around each of the industrial facility. In each sampling point, six samples of soil and plant material of given species at intervals of 2–3 m were collected separately, from which average laboratory samples were prepared. The crop collection from the neighborhood of the Głogów smelter included thirty samples wheat (*Triticum* L.). Thirty samples of oilseed rape (*Brassica napus* L.) were collected from around the Legnica smelter. The plant species harvested close to the Oława smelter and the Żelazny Most tailings pond included thirty samples of *Taraxacum officinale* (F.H. Wigg.) and thirty samples of grasses without specifying the particular species. Air-dried samples of soils were sieved through a sieve with less than 1 mm meshes. The aerial parts of plants (biomass) were washed, dried, and then crushed with mortar.

The preparation of samples for the elemental analysis using atomic absorption spectroscopy included weighing approx. 0.2 g of homogeneous dry material (soil or plant) and digestion in nitric acid of 65% (8 ml) according to PN-ISO 11465 (1999). The mineralization process was conducted in a closed Microwave Digestion System using Start D Milestone equipment, in conditions of a linear temperature increase of 220 °C with the use of microwave power of 800 W. The measurements of zinc, copper, and lead content were performed through the FAAS and the GFAAS instrumental method using the Thermo Solaar iCE 3500 device (PN-ISO 11047, 2001). In the trace metal analysis were applied the certified reference materials (CRM) from Sigma Aldrich. The reagent blank samples were used to check the instrument readings. The limits of detection were estimated based on three times the standard deviation for digestion blanks. The accuracy of the determination was controlled using the method of the standard addition. The percentage of the recovery was 94–98%. The pH and the electrical conductivity measurements in soils with a stoichiometry of 1:2.5 (m:v) were conducted according to PN-ISO 10390 (1997).

The AAS measurement results were verified through the standard deviation, the coefficient of variation, and the confidence interval. The accepted results of the coefficient of variation for a given point were within 10%. Statistical analysis was made based on the *t* Student test with the number of degrees of freedom equalling 5 and the *p* value of 0.05. Chemical analyses have been carried out in the certified Laboratory of Toxicology and Environmental Research in the Faculty of Environmental Engineering at the Wrocław University of Science and Technology.

The content of metals in soil and plant was calculated as the average value with the standard deviation for six independent environmental samples from one location. The average values of pollution index for a given location were used.

The statistical analysis was carried out using the program Statistica ver. 13.1 and Microsoft Excel 2013. The data was checked for normal distribution (Shapiro-Wilk’s *W* test). Tests of significance were made at 95% confidence level. The Mann-Whitney *U* test was used for the data which did not show normal distribution. Spearman’s rank correlation coefficients were calculated for elemental concentrations for all samples. Correlations were considered strong when higher than 0.7, and *p* value was 0.05.

### Pollution indices

The contamination factor (${C}_f^i$) defines the ratio of the mean content of metal in soil from six sampling sites (${C}_{0-1}^i$) to the preindustrial concentration of individual metal (${C}_n^i$) (Hakanson 1980). In our work, we applied the concentration of elements in the Earth’s crust as a reference value similarly to Loska et al. (2004). The average content of copper, zinc, lead, and cadmium in the Earth’s crust is respectively, in mg/kg: 39 (Cu), 67 (Zn), 17 (Pb) (Tylor and McLennan 1995).

**Table:** 

$$ {C}_f^i=\frac{C_{0-1}^i}{C_n^i} $$	
${C}_f^i$value	Soil quality
${C}_f^i$ < 1	Low contamination
1 <${C}_f^i$ < 3	Moderate contamination
3 <${C}_f^i$ < 6	Considerable contamination
6 <${C}_f^i$	Very high contamination

The degree of contamination in soil (*C*_*d*_) is defined as a sum of contamination factors for all trace elements (${C}_f^i$). Four classes of the *C*_*d*_ parameter were categorized by Hakanson (1980). Zahran et al. (2015) introduced a modification of earlier defined ranges of the degree of contamination, assuming as *n* the number of trace elements to be determined.

**Table:** 

$$ {C}_d=\sum \limits_{i=1}^{i=n}{C}_f^i $$		
*C*_*d*_ value (Zahran et al. 2015)	*C*_*d*_ value (Hakanson, 1980)	Soil quality
*C*_*d*_ < *n*	*C*_*d*_ < 8	Low degree of contamination
*n*<*C*_*d*_ < 2*n*	8 <*C*_*d*_ < 16	Moderate degree of contamination
2*n*<*C*_*d*_ < 4*n*	16 <*C*_*d*_ < 32	Cconsiderable degree of contamination
4*n*<*C*_*d*_	32 <*C*_*d*_	Very high degree of contamination

According to Abrahim and Parker (2008), Machender et al. (2011), and Rahman et al. (2012), the modified degree of contamination (*mC*_*d*_) is the average value of pollution indices for all trace elements (${C}_f^i$), provided that at least three chemical elements (*n*) are used in the calculations.

**Table:** 

$$ {mC}_d=\frac{C_d}{n} $$	
*mC*_*d*_ value	Soil quality
*mC*_*d*_ < 1.5	Nil to very low degree of contamination
1.5 <*mC*_*d*_ < 2	Low degree of contamination
2 <*mC*_*d*_ < 4	Moderate degree of contamination
4 <*mC*_*d*_ < 8	High degree of contamination
8 <*mC*_*d*_ < 16	Very high degree of contamination
16 <*mC*_*d*_ < 32	Extremely high degree of contamination
32 <*mC*_*d*_	Ultra high degree of contamination

The pollution load index (*PLI*) is the geometric average of the impurity coefficients (${C}_f^i$) and determines the contribution of all metals in a given place (Tomlinson et al. 1980; Jorfi et al. 2017). This parameter allows to assess the level of environmental contamination in order to undertake monitoring or repair activities aimed at improving soil quality.

**Table:** 

$$ PLI=\sqrt[n]{C_{f1}\cdotp {C}_{f2}\cdotp \dots \cdotp {C}_{fn}} $$	
*PLI* value	Soil quality (Jorfi et al., 2017)
0 < *PLI* < 1	Unpolluted
1 < *PLI* < 2	Moderately polluted to unpolluted
2 < *PLI* < 3	Moderately polluted
3 < *PLI* < 4	Moderately to highly polluted
4 < *PLI* < 5	Highly polluted
5 < *PLI*	Very highly polluted

The index of geoaccumulation (*I*_*geo*_*)* applies a logarithm operation of the data set and the constant equal 1.5, which allows for the elimination of possible differences in the content of the studied element in the soil due to the possible variation in the background (*B*_*n*_) (Stoffers et al. 1986; Ruiz 2001) and a small influence of anthropogenic activity (Muller 1981). In this work employed a modified *I*_*geo*_ index to make calculations because the background value was denoted of the concentration of trace elements in the Earth’s crust (Tylor and McLennan 1995).

**Table:** 

$$ {I}_{geo}={\log}_2\frac{C_n^i}{1.5{B}_n} $$		
*I*_*geo*_ class	*I*_*geo*_ value	Soil quality
0	*I*_*geo*_ ≤ 0	Practically uncontaminated
1	0 < *I*_*geo*_ < 1	Uncontaminated to moderately contaminated
2	1 < *I*_*geo*_ < 2	Moderately contaminated
3	2 < *I*_*geo*_ < 3	Moderately to heavily contaminated
4	3 < *I*_*geo*_ < 4	Heavily contaminated
5	4 < *I*_*geo*_ < 5	Heavily to extremely contaminated
6	5 < *I*_*geo*_	Extremely contaminated

In the worldwide studies, there is a lack of consistency in the application of geochemical background values (unification) and the selection of a standardized (universal) indicator describing the contamination of soils. The diversity in the ways of assessing the degree of soil contamination makes it impossible to compare soils around industrial facilities, even with a similar kind of production. During the work, we have chosen indices: *I*_*geo*_ and C_*f*_ with the reference background, and *C*_*d*_/*mC*_*d*_ and *PLI*, which made it possible to easily compare them with each other, which is confirmed statistically by the linkage distances between clusters according to Kowalska et al. (2018). So far, no comprehensive analysis of the environment with the use of pollution indices around non-ferrous metallurgy works and tailings pond with metal ore post-flotation waste has been carried out. Therefore, in this work, an effort was made to look at the indices of pollution in the assessment of soil quality. We point out that environmental pollution indices are a useful tool in assessing the quality of soils. In contrast, elemental concentrations inform about the exceeding of the permissible standards, without taking into account the geochemical level of the element in soil and not distinguishing between the degrees of soil pollution.

In order to monitor the content of these elements in plants, the bioaccumulation coefficient (*BAC*) is used. This parameter compares the concentration of the trace element in biomass (plants aboveground parts, ${P}_t^i$) with its content in the soil (${C}_{0-1}^i$). It further enables categorization of plants as accumulators (*BAC* > 1) or excluders (*BAC* < 1) of trace elements (Olowoyo et al. 2010).

$$ BAC=\frac{P_t^i}{C_{0-1}^i} $$

The assessment of total accumulation of all trace elements in plants is based on the metals accumulation index of *MAI* (Liu et al. 2007), where *n* is the total number of trace elements to be determined and *I*_*i*_ is the subindex for variable *i*. The parameter *I*_*i*_ is obtained by dividing the average concentration value of each metal in biomass (${P}_t^i$) by the standard deviation (${\delta P}_t^i$).

$$ MAI=\left(\frac{1}{n}\right)\sum \limits_{i=1}^{i=n}{I}_i;{I}_i=\frac{P_t^i}{{\delta P}_t^i} $$

The comprehensive bioconcentration index (*CBCI*) is based on fuzzy synthetic and allows to assess the overall performance of plant species in the phytoremediation of soils contaminated with many trace elements (Zhao et al., 2014).

$$ CBCI=\left(\frac{1}{n}\right)\sum \limits_{i=1}^{i=n}{\upmu}_i;{\upmu}_i=\left\{\begin{array}{c}0\kern3em BAC={BAC}_{\mathrm{min}}\\ {}\frac{BAC-{BAC}_{\mathrm{min}}}{BAC_{\mathrm{max}}-{BAC}_{\mathrm{min}}},{BAC}_{\mathrm{min}}< BAC<{BAC}_{\mathrm{max}}\\ {}1\kern3em BAC={BAC}_{\mathrm{max}}\ \end{array}\right. $$

## Results and discussion

The studied soils were mainly slightly acidic (pH 5.6–6.5) and neutral (pH 6.6–7.2), which is confirmed by the data from Medyńska-Juraszek and Kabała (2012 and 2010), Kabała et al. (2008), and Cuske et al. (2013). No alkaline soils were found near the copper smelters in Legnica and Głogów. Soils around all facilities were poorly salted. The electrical conductivity was basically below 25 mS/m (92% of samples in Żelazny Most, 75% in Głogów, 100% in Legnica, and 67% in Oława).

### Trace element contents

The content of trace elements in the top layer of soils around the smelting works and the Żelazny Most copper ore tailings pond is presented in Table 1. A high value of the coefficient of variation (*CV* > 0.90) means high extent of spatial variation and indicates high degree of anthropogenic contribution (point pollution). Other *CV* values, except for copper in Oława, also indicate that studied areas have undergone anthropogenic influence with moderate level of spatial inhomogeneous. Such results can also be achieved from the *p* value of the normal distribution test. All elements have *p* values below 0.05, which implies that they cannot pass the normal distribution test. It may also suggest that the elements have anthropogenic source. When the data did not show normal distribution, the Mann-Whitney *U* test was used and showed that the differences between the medians of the concentrations of two elements are statistically significant (*p* < 0.05).

**Table 1:** Descriptive statistics of metals in soils (mg/kg)

Metal	Range	Avg.	SD	Q_1_	Med.	Q_3_	Skewness	Kurtosis	*CV*
Żelazny Most, *n* = 30									
Zn	32.78–92.97	48.28	17.98	34.76	38.47	55.81	1.28	0.71	0.37
Cu	36.47–337.11	93.61	90.50	40.47	51.92	84.04	1.80	2.09	0.97
Pb	2.71–82.93	19.67	21.03	9.62	13.64	17.24	2.30	4.64	1.07
Głogów, *n* = 30									
Zn	29.75–836.03	124.28	215.04	42.93	58.73	78.76	3.02	7.48	1.73
Cu	23.56–250.97	130.12	69.49	72.80	118.00	186.35	0.20	− 1.29	0.53
Pb	10.44–89.52	43.49	21.09	28.00	37.32	53.78	0.82	− 0.30	0.48
Legnica, *n* = 30									
Zn	24.33–241.52	59.46	54.70	31.31	37.93	72.86	2.63	6.28	0.92
Cu	51.18–2476.30	290.38	559.62	84.27	102.86	118.53	3.11	9.24	1.93
Pb	50.00–685.77	124.92	149.97	66.06	80.98	102.10	3.31	10.14	1.20
Oława, *n* = 30									
Zn	190.81–2535.63	825.70	617.48	270.15	717.32	1024.57	1.30	1.64	0.75
Cu	28.89–44.79	36.51	4.44	33.19	36.50	39.81	0.06	− 1.12	0.12
Pb	90.34–1168.51	329.02	305.77	120.92	161.04	405.26	1.45	0.99	0.93

The positive values of skewness mean that most of the results are below the average value, whereas the positive values of kurtosis inform about the existence of a large pool of results close to the average value and at the same time a small number of outcomes with the extreme values. For the studied areas, the arithmetic average value is significant influenced by the single very high concentrations of a given trace element. A special situation occurs in Legnica, where the largest spread between the concentrations of a given metal was observed, with a small arithmetic average value at the same time. The maximum concentrations of trace elements were greater than the average values of 8.5-fold for copper and 6-fold for lead. In Głogów, a large spread of results for zinc concentrations in soil samples was observed. The maximum value was almost 7 times higher than the average value. A large dispersion of results for zinc was observed in Oława as well. The average value was 3 times lower than the maximum value, but at the same time similar to the median.

The values of Spearman’s rank correlation coefficient (*p* value of 0.05) indicate strong correlation between Cu and Pb (0.73) and moderate correlation between Zn and Pb (0.59) in Głogów. Moderate correlations were also recorded for pairs of Cu-Pb (0.54) and Zn-Pb (0.59) in Oława, for pairs of Cu-Zn (0.66) and Cu-Pb (0.42) in Legnica, and also between Cu and Zn in Żelazny Most (0.69), which may suggest the metals originated from industrial activity.

When comparing obtained concentrations of metals in soils with the literature data from previous years, from 5 to 10 years back for studied industrial facilities (Table 2), no significant reduction of metals content in the top of soil profile is still observed. High levels of metals in topsoils within a distance of up to 2.5 km from the studied facilities are noticed. For other facilities in the world with similar activities, the comparable and sometimes even much higher concentrations of trace elements than in Poland were observed (Ettler 2016).

**Table 2:** Ranges of metal contents in topsoil around smelting plants and tailings pond (mg/kg)

Cu	Pb	Zn	Location	References
Copper smelter				
29–3043	63–1955	191–607	Reda, Russia	Vorobeichik and Kaigorodova, 2017
220–2540	40–230	–	Bor, Serbia	Nikolić et al., 2011
303–9590	–	140–4020	Legnica, Poland	Medyńska-Juraszek and Kabała, 2010
58–455	66–379	–	Głogów, Poland	Kabała et al., 2008
9–1597	10–295	14–180	Port Kembla, Australia	Martley et al., 2004
113–1890	11–75	32–146	Cliff Smelter, Sudbury, Canada	Adamo et al., 2002
11–403	8–57	13–144	Falconbridge Smelter, Sudbury, Canada	Adamo et al., 2002
112–1330	8–88	43–67	Conistone Smelter, Sudbury, Canada	Adamo et al., 2002
Zinc/zinc-lead smelter				
-	300–9600	400–26,000	Olkusz, Poland	Kapusta and Sobczyk, 2015
11–88	55–1894	196–2404	Oława, Poland	Cuske et al., 2013
27–2550	174–15,800	462–9650	Avonmouth, UK	Filzek et al., 2004
14–106	47–1279	55–1632	Trail, Canada	Goodarzi et al., 2002
17–34	113–306	258–2167	Auby, France	Sterckeman et al., 2002
16–46	101–1132	150–1378	Noyelles-Gdt, France	Sterckeman et al., 2002
-	38–5410	82–31,175	Auby and Noyelles-Gdt, France	Sterckeman et al., 2000
Post-flotation tailings pond				
9–678	22–213	15–72	Żelazny Most (Cu ore), Poland	Medyńska-Juraszek and Kabała, 2012
440–3800	21–1700	40–890	Bor (Zn-Pb ore), Serbia	Antonijević et al., 2012
-	172–5530	378–13,498	Bukowno (Zn-Pb ore), Poland	Cabala et al., 2009
2–99	56–2696	50–10,638	Bukowno (Zn-Pb ore), Poland	Krzaklewski et al., 2004

### Pollution indices and soil quality

The C_*f*_ index values usually suggest a higher degree of soil contamination with metals in comparison with the *I*_*geo*_ index. Applying the logarithmic transformation of the initial data narrows the *I*_*geo*_ index compartments down to the range between the minimum and maximum values for a given population of results. For this reason, the *I*_*geo*_ index makes a distinction between a bigger number of different degrees of soil contamination. It allows to verify the quality of soils without classifying them equally. This is particularly important in the case of heavily contaminated soils around industrial facilities. However, the C_*f*_ index provides the basis for the calculation of complex indices group and the degree of soil pollution with all analyzed heavy metals. Shapiro-Wilk’s *W* test conducted for the C_*f*_ and the *I*_*geo*_ indices confirmed the lack of normal distribution (*p* < 0.05). The Mann-Whitney *U* test showed that the differences between the C_*f*_ and *I*_*geo*_ indices are statistically significant (*p* < 0.05) with the exception of the Zn-Pb pair in Żelazny Most (*p* value of 0.16).

#### Żelazny Most tailings pond

**Fig. 1:**
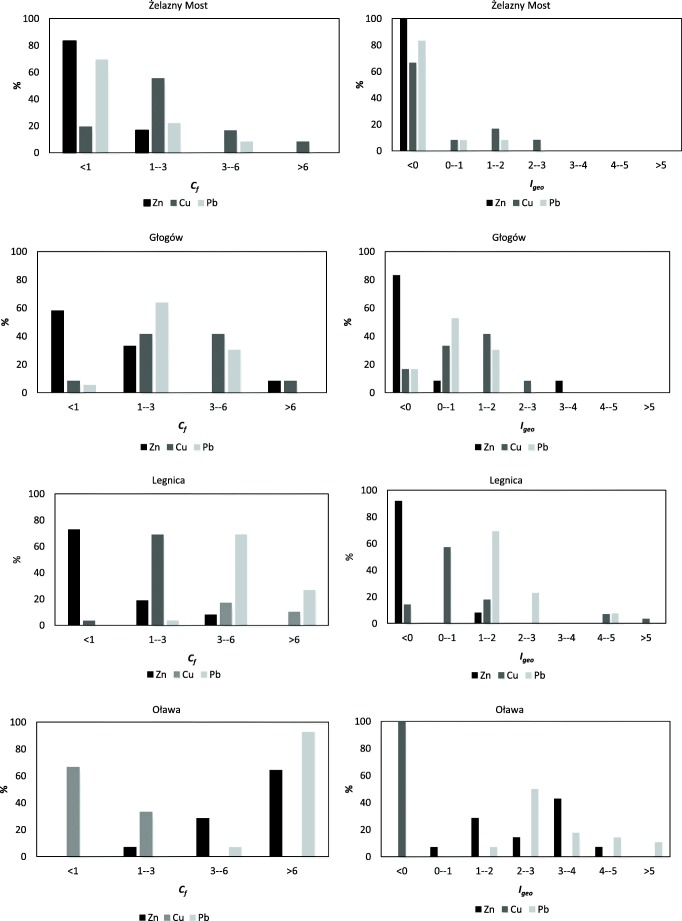
Percentage of samples in the C_*f*_ and *I*_*geo*_ classes

The median of *I*_*geo*_ for analyzed metals does not indicate contamination of soils around the Żelazny Most reservoir (Table 3). Despite that fact, the median of C_*f*_ shows moderately contaminated soils with copper because some sampling point soils are heavily or considerable contaminated with this element (Fig. 1).

**Table 3:** Descriptive statistics of pollution indices in soils

	Avg.	SD	Med.	Q1	Q3	Avg	SD	Med.	Q1	Q3
Żelazny Most	C_*f*_					*I*_*geo*_				
Zn	0.72	0.27	0.57	0.52	0.83	− 1.14	0.47	− 1.39	− 1.53	− 0.86
Cu	2.40	2.33	1.33	1.04	2.15	0.23	1.04	− 0.18	− 0.53	0.41
Pb	1.16	1.24	0.80	0.57	1.01	− 0.92	1.21	− 0.90	− 1.43	− 0.57
*C*_*d*_	4.28	3.57	2.63	2.31	4.22	-				
*mC*_*d*_	1.40	1.20	0.87	0.77	1.34	*2.45/3.61				
*PLI*	1.15	0.81	0.82	0.73	1.04	*2.11/3.47				
Głogów	C_*f*_					*I*_*geo*_				
Zn	1.85	3.21	0.88	0.64	1.18	− 0.52	1.20	− 0.78	− 1.23	− 0.35
Cu	3.34	1.78	3.03	1.87	4.78	0.89	0.94	1.00	0.32	1.67
Pb	2.56	1.24	2.20	1.65	3.16	0.60	0.71	0.55	0.13	1.08
*C*_*d*_	7.75	4.67	6.52	3.83	9.51	-				
*mC*_*d*_	2.58	1.56	2.17	1.28	3.17	*1.31/1.14				
*PLI*	2.16	1.24	1.98	1.17	2.52	*1.45/1.62				
Legnica	C_*f*_					*I*_*geo*_				
Zn	1.05	1.02	0.54	0.48	0.98	− 0.97	1.02	− 1.48	− 1.66	− 0.66
Cu	9.13	15.40	2.80	2.19	4.29	1.38	1.54	0.90	0.54	1.44
Pb	9.69	11.42	5.41	4.12	6.64	2.30	1.09	2.02	1.63	2.31
*C*_*d*_	13.37	20.26	7.45	5.82	9.55	-				
*mC*_*d*_	4.46	6.75	2.48	1.94	3.18	*2.86/7.06				
*PLI*	3.09	4.31	1.83	1.43	2.30	*2.92/7.86				
Oława	C_*f*_					*I*_*geo*_				
Zn	12.32	9.22	10.71	4.03	15.29	2.63	1.12	2.88	1.43	3.35
Cu	0.94	0.11	0.94	0.85	1.02	− 0.69	0.18	− 0.68	− 0.82	− 0.56
Pb	19.35	17.99	9.47	7.11	23.84	3.19	1.15	2.66	2.25	3.99
*C*_*d*_	32.61	22.91	23.00	11.85	53.66	-				
*mC*_*d*_	10.87	7.64	7.67	3.95	17.89	*0.76/− 0.69				
*PLI*	5.54	2.69	4.63	2.81	8.43	*0.39/− 1.46				

*Skewness/kurtosis

#### Głogów copper smelter

According to the *I*_*geo*_ classification, 50% of the population of samples are moderately contaminated with copper, with division into the 2nd class and the 3rd class (Fig. 1). The median of C_*f*_ for copper confirms enrichment of soils (Table 3). For zinc and lead, the medians of the *I*_*geo*_ indices do not indicate contamination of soils. The average of C_*f*_ for zinc is 2 times higher than median of C_*f*_ which confirms the heterogeneity of the distribution of metal content in soils.

#### Legnica copper smelter

The median of *I*_*geo*_ does not indicate contamination of soils with copper (Table 3); however, the median of C_*f*_ shows moderate contamination of soils. The average of C_*f*_ is much higher than the median value because very highly contaminated soils with copper are noticed in the border area of the smelter at a distance below 100 m from the facility border (5th and 6th classes of *I*_*geo*_) (Fig. 1).

The median of *I*_*geo*_ index for lead classifies soils as moderately polluted. Soils closer to the smelter belong to the 5th class of *I*_*geo*_ (heavily to extremely contaminated). The median of C_*f*_ confirms a considerable contamination of soils with lead.

According to the median of *I*_*geo*_ and C_*f*_ indices, zinc is not a pollutant of soils. Only in the border area of the smelter a moderate pollution of soils with zinc was recorded (2nd class of *I*_*geo*_).

#### Oława zinc smelter

The medians of *I*_*geo*_ for zinc and lead show accumulation of metals in soils in the immediate vicinity of the Oława smelter. The *I*_*geo*_ classification allows to assign the soils into classes between the 3rd and the 5th for zinc and from the 3rd up to the 6th class of soils for lead (Fig. 1). High medians of C_*f*_ for both metals (Table 3) indicate very high or considerably contamination of soils. Copper is not a pollutant of soils around the Oława smelter.

Kabir et al. (2012) presented the values of the *I*_*geo*_ index for the zinc smelters in China, Slovenia, and the UK. Compared with our results, the indicated areas were more lead-polluted (heavily to extremely contaminated): 4.85 Slovenia; 3.98 China; 2.18 the UK. The *I*_*geo*_ value of zinc at China smelter (2.67) was similar to the values in Oława zinc smelter. In Slovenia, high level of copper contamination was recorded (3.39) which was not noticed around the Oława smelter.

The soil quality near the copper smelter in Albania (Rubik) shows extreme soil contamination with copper (4.24) and zinc (4.63) according to Shallari et al. (1998). The *I*_*geo*_ index for lead of 2.40 was close to the value measured for Legnica smelter in Poland. The area around the copper smelter in Spain was moderately contaminated with lead (1.50) according to Kabir et al. (2012).

#### Total level of soil pollution

According to Zahran’s et al.'s classification (2015), the sum of the pollution indices for all trace elements (med_*C*_*d*_) indicates a very high degree of the pollution of soils around zinc smelter and considerable degree of pollution around both copper smelters (Table 3). Particularly high *C*_*d*_ values were recorded for the area around the Oława smelter (73% of samples). At the Głogów and Legnica copper smelters, the value of *C*_*d*_ exceeded the limit of 12 (ca. 16% of samples population). The degree of the contamination (med_*C*_*d*_) of soils around the Żelazny Most reservoir is low for studied chemical elements.

According to Hakanson’s classification (1980), studied soils have low degree of contamination (med_*C*_*d*_) close to the copper smelters of Legnica and Głogów and the tailing pond of Żelazny Most. In the Oława smelter, 43% of soil samples were identified as very highly polluted (Table 3).

The percentage shares of individual metals in contaminated soils are presented as the ratio of C_*f*_ to *C*_*d*_ indices. The soils enrichment with metals near the copper smelters and the Cu post-flotation tailings pond was shown in descending order: Legnica Pb (49%) > Cu (46%) > Zn (5%), Głogów Cu (46%) > Pb (33%) > Zn (24%), and Żelazny Most Cu (56%) > Pb (27%) > Zn (17%). Around the zinc smelter in Oława trace elements comprised the following order: Pb > Zn > Cu. The share of lead and zinc in soils in Oława was significant and amounted to, respectively, 59% and 38%.

The medians of *mC*_*d*_ indicate lack of soil contamination for Żelazny Most, moderate degree of soil contamination for Legnica and Głogów, and high degree in Oława. The soil quality assessed with the use of *PLI* index coincides with the assessment of contamination degree of soils using *C*_*d*_ median (Table 3). Thus, all indices of *C*_*d*_, *mC*_*d*_*,* and *PLI* similarly determine the quality of soil in the investigated areas.

**Fig. 2:**
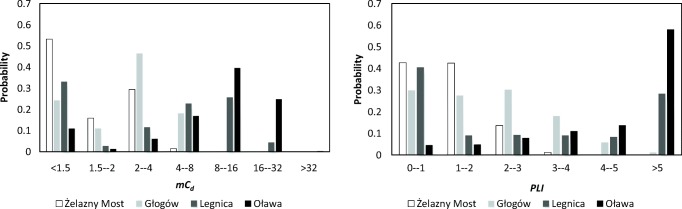
Distribution of metals in soils

A large asymmetry in the frequency distribution of *mC*_*d*_ and *PLI* was observed in locations connected with copper smelting and tailing pond. Positive skewness values of the right-skewness distribution confirm the predominance of soils with low contamination for the studied areas (Table 3). At the Oława smelter, the soils are very heavily contaminated with metals and there are few results close to the average (negative kurtosis) (Fig. 2).

Due to a similar type of industrial activity (copper ore processing) and the use of the individual (C_*f*_) and complex (*C*_*d*_, *mC*_*d*_, *PLI*) contamination indices by Demková et al. (2016), we can compare the level of contamination of studied areas in Poland with the area of mining and processing of copper and mercury in Central Spiš in Slovak Republic. The authors used the contamination indices to compare the soil pollution degree in 1997 and 2015. Despite extremely high pollution, an improvement in the quality of soil was noticed after 18 years when the mining activity was stopped and the processing activity of copper was limited at the beginning of the twenty-first century. Comparison of the complex contamination indices for three trace elements (Cu, Zn, Pb) shows similar degree of soils contamination in Oława (Table 3) and in Spiš, Slovakia for 2015 (Avg_*C*_*d*_ 30.5, *mC*_*d*_ 10.2, *PLI* 8.38). In the Legnica-Głogów Copper District area in Poland, the values of these indicators are much lower: 7 times in Żelazny Most, 4 times in Głogów, and 2 times in Legnica. The lower pollution of soils can be attributed to conducting the study outside of the border industrial plants.

In Poland, the first indices analysis of soils’ quality for the comprehensive evaluation of the degree of soil contamination in the industrial area was performed by Loska et al. (2004). The Upper Silesian Industrial Region of Poland is an area exposed to the emission of pollutants from the main industrial center of Poland, the Trzyniec steel smelter in the Czech Republic, and local coal mining. Analysis of the *C*_*d*_, *mC*_*d*_, and *PLI* indices for the three elements does not show contamination of soils with lead, zinc, and copper. The higher values of parameters (Table 3) for copper and zinc smelters we studied indicate the impact of industrial activity on the soil environment in the immediate vicinity of these industrial plants.

### Plants’ response to the quality of soils

The cereal plants are grown within a radius of up to 2.5 km from the copper smelters under consideration. The dominant species is *Triticum* L. in Głogów and *Brassica napus* L. in Legnica. The area around the Żelazny Most post-flotation tailings pond from the copper industry is significantly wooded, that is why grasses without specifying of the species and *Taraxacum officinale* were picked up in the foreground of the tailings pond. The Oława smelter is located within the city; therefore, the same plants were collected from the green areas as from the area of Żelazny Most.

**Fig. 3:**
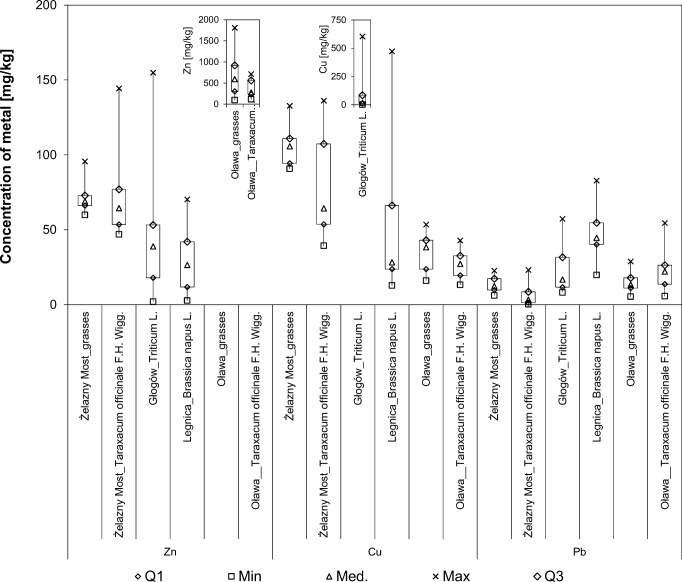
Average content of trace elements in plants (*n* = 30)

Metal concentrations observed in biomass in the studied areas show high levels of trace elements close to the industrial facility and a decrease in metal concentrations along with the increase of the distance from its borders (the lack of normal distribution, *p* < 0.05 and statistically significant differences between medians of concentrations, *p* < 0.05). Particularly high levels of trace elements in biomass (Fig. 3) were observed in Oława. The median of zinc in grasses and *Taraxacum officinale* are almost 9 times and 4 times higher as in the analogous plant biomass in Żelazny Most. Lead is almost 7 times higher in *Taraxacum officinale* in Oława. Copper values are over 2 times higher in the plant biomass in Żelazny Most.

Zinc values in the cereal biomass of *Triticum* L. in Głogów are 1.5 times higher than in *Brassica napus* L. in Legnica, although more copper (1.5 times) and lead (2.5 times) in *Brassica napus* L. were observed.

The values of the *MAI* index for the *Taraxacum officinale* demonstrate this species’ good capability to adsorb trace elements in biomass from the contaminated soils.

Trace elements’ uptake in plants (*MAI*) was the highest around the Żelazny Most tailing pond, with 52.05 for grasses and 192.86 for *Taraxacum officinale*. Around the Oława smelter, 50.92 and 65.71 were reported, respectively, for the same plants. The lower values of the *MAI* index were obtained for the cereal plants of *Triticum* L. in Głogów (29.93) and *Brassica napus* L. in Legnica (10.54).

Giacomino et al. (2016) and Levei et al. (2017) obtained low of the *MAI* values of 1.61–2.25 for *Taraxacum officinale* in the traffic-impacted areas in Italy (Cuneo province in Piedmont) and of 1.4 for the mine tailings in the Metaliferi Mountains in Baia Mare County in Romania. Much higher *MAI* values in *Taraxacum officinale* were presented by Nadgórska-Socha et al. (2017) from Poland. The values of metal accumulation indices ranged from 4.68 to 28.1 for the area connected with the industry emitters (iron smelter, coking plant, and waste processing plant) in the Dąbrowa Górnicza city in the Upper Silesia.

Strong and very strong degrees of positive correlations between metal content in soil and metal content in plant are observed for copper: grasses_Żelazny Most (0.89), *Triticum* L._Głogów (0.60); for zinc: *Taraxacum officinale*_Żelazny Most (0.76); and for lead: grasses_Żelazny Most (0.73), *Triticum* L._Głogów (0.91). Other correlation coefficients confirm moderate or weak relationship.

**Fig. 4:**
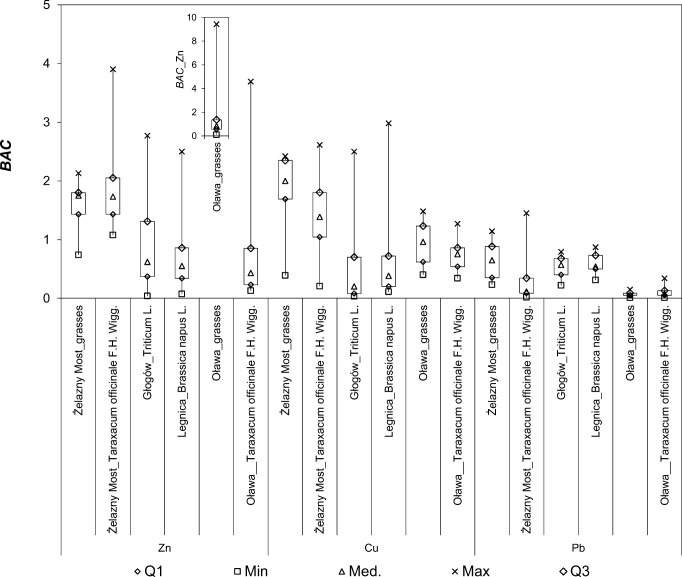
Bioaccumulation of metals in plant

The activity of industrial facilities may affect the content of metals in biomass in a reason of the mobility of trace elements from the soil solution. If the *BAC* values are higher than the unit, the plants are classified as hyperaccumulators, while if the *BAC* values are lower than the unit, plants are considered to be tolerant in relation to the trace element (Antonijević et al. 2012). Based on this principle, *Taraxacum officinale* and grasses can be considered good indicators of metals. The bioaccumulation of zinc and copper was observed for *Taraxacum officinale* and grasses in Żelazny Most and for grasses in Oława (Fig. 4). The bioaccumulation of the abovementioned metals in grasses was greater than in *Taraxacum officinale*. In addition, strong correlations between the bioaccumulation and the concentration of metals in soil were observed in Żelazny Most (grasses, Cu and Zn; *Taraxacum officinale*. Cu) and Oława (grasses and *Taraxacum officinale*, Zn).

In the cereal plants, no bioaccumulation of metals in biomass was observed. For *Triticum* L. in Głogów, bioaccumulation of zinc was reported in 25% (upper quartile) of the samples population. In Legnica, median values of the *BAC* index do not indicate an increasing level of metals in the biomass of *Brassica napus* L. The soils in the vicinity of the Legnica and Głogów copper smelters may be used for cultivation of cereal crops due to low bioaccumulation of studied trace elements.

Despite the observed bioaccumulation of individual trace elements in *Taraxacum officinale* and grasses, the multi-metal accumulation is not observed in tested plants at the same time (*CBCI* of 0.28 to 0.57).

In the leaves of *Taraxacum officinale* within the city of Dąbrowa Górnicza in the industrial region of Upper Silesia in Poland, the average value of the *BAC* index for zinc was 2.36 (Nadgórska-Socha et al. 2017). It was considered that *Taraxacum officinale* is suitable for using in areas with significant soil and air pollution, especially with trace elements.

In the plant biomass from the Głogów copper smelter and the Żelazny Most copper ore tailings pond, strong correlations of copper with zinc: 0.87 for *Triticum* L./0.82 grasses/0.93 *Taraxacum officinale*, and also correlations of copper with lead: 0.56/0.53/0.91, were observed. At the same locations, a positive moderate correlation of zinc with lead (0.51/0.53/0.75) was also noticed. In Oława, moderate correlation was noted for copper and lead in grasses (0.59).

## Conclusions

The worldwide research publications mainly show that the contents of trace elements in soils vary along the distance from the site or the depth in the soil profile. Therefore, comparing the environment around objects with similar industrial activity is often impossible due to different permissible content of a given element in soil, applicable in a regulation of a given country. The advantage of using pollution indicators is the possibility of comparing different objects in the world directly, provided that the reference geochemical background values are used. Thus, the indicators provide information directly on the quality of the soil environment. For objects associated with the smelting of colored metals, there is still a lack of information on the quality of the soil environment based on pollution indices.

The pollution indices are an objective tool for assessing the real enrichment of soils with trace elements. The individual indices were used to obtain information on the level of soil pollution by each of the analyzed metals. The complex indices were used to determine the total pollution of the examined areas. The simultaneous use of several indicators allows us to more accurately assess the pollution of soil with heavy metals.

The use of the C_*f*_ indicator resulted in the soil being classified as very contaminated with copper and lead in Legnica and zinc and lead in Oława. Thanks to use of the *I*_*geo*_ parameter, other classes—from 2nd to even 6th—have been assigned, specifying degrees of soil pollution from moderately to heavily contaminated. And so the assessment of soil quality using the *I*_*geo*_ index allows to identify soils with less pollution as compared with assigning soils to a common C_*f*_ class.

The quality of soils within a distance of up to 2.5 km from the studied facilities is poor due to the high values of pollution indices for trace elements: copper and lead in the Legnica smelter and in some sampling points at the Głogów smelter, and lead and zinc in the Oława smelter. The complex indices integrate and average all available analytical data and allow comparison of the degree of general pollution in different places. The complex indices of *C*_*d*_, *mC*_*d*_, and *PLI* assessed the soil around the Oława smelter as very highly contaminated with trace elements. Soils around copper smelters were moderately contaminated with metals. The individual and complex pollution indices do not indicate contamination of soils with copper, zinc, and lead around the Żelazny Most reservoir.

The research on accumulation of metals in biomass with the use of the *BAC* and the *MAI* indices shows that grasses and *Taraxacum officinale* accumulate larger amounts of trace elements as compared with the biomass of the cereal plants. Additionally, strong correlations between the content of the trace element in the soil and the amount of metal in grasses and *Taraxacum officinale* were recorded. The multielemental bioaccumulation in plants around the examined industrial facilities was not observed. Cereals can be harvested from the areas of the Głogów and Legnica smelters, but with periodic control of the trace elements content in biomass.
